# Removal of diclofenac from aqueous solutions by adsorption on thermo-plasma expanded graphite

**DOI:** 10.1038/s41598-021-83117-z

**Published:** 2021-02-09

**Authors:** Marco Cuccarese, Sergio Brutti, Angela De Bonis, Roberto Teghil, Ignazio Marcello Mancini, Salvatore Masi, Donatella Caniani

**Affiliations:** 1 Scuola di Ingegneria, Università degli Studi della Basilicata, viale dell’Ateneo Lucano n.10, 85100 Potenza, Italy; 2 Dipartimento di Chimica, Università di Roma “La Sapienza”, Piazzale Aldo Moro 5, 00185 Roma, Italy; 3 Dipartimento di Scienze, Università degli Studi della Basilicata, viale dell’Ateneo Lucano n.10, 85100 Potenza, Italy

**Keywords:** Environmental sciences, Chemistry, Materials science

## Abstract

The adsorption of diclofenac on thermo-plasma expanded graphite (a commercial product) from water solutions was investigated. The adsorbent material was characterized by SEM, TEM, BET, Raman and X-ray diffraction analyses. Typical diffractogram and Raman spectrum of graphitic material, dimension of 24.02 nm as crystallite dimension and a surface area of 47 m^2^ g^−1^ were obtained. The effect of pH on the adsorption capacity was evaluated in the range 1–7 and the adsorption mechanism was described by kinetic and isothermal studies. Pseudo-second order and Dubinin–Radushkevich models agreed with theoretical values of adsorption capacity (i.e. 400 and 433 mg g^−1^, respectively) and resulted to be the best fit for kinetics and isothermal experimental data. The thermodynamics of the process was evaluated by plotting the adsorption capacity/concentration ratio at the equilibrium as a function of different values of the multiplicative inverse of temperature. Moreover, the adsorbent regeneration was also investigated, comparing two different remediation techniques. Solvent washing performed with NaOH 0.2 M and thermo-treatment carried out by heating in an oven at 105 °C for 2 h and then at 200 °C for 4 h. The thermo-treatment was the best technique to regenerate the adsorbent, ensuring same performance after 4 cycles of use and regeneration.

## Introduction

Surface water contamination is a growing concern and different kinds of contaminants and remediation techniques have been widely investigated in literature. Pharmaceuticals and Personal Care Products (PPCPs) have increasingly been found in surface water and, even though they are normally present at very low concentrations, several scientific studies^1–4^ have proved their negative impact on aquatic life. Conventional wastewater treatment plants cannot completely remove PPCPs because of their high polarity and solubility in water^5–8^. These characteristics represent the main challenge for the removal of PPCPs from water solution. Different methods, such as photodegradation^9^, coagulation-flocculation^10^, biodegradation^11^, chlorination^12^ and ozonation^13^, have been used to remove PPCPs by trapping or degradation. Coagulation-flocculation methods usually present a removal percentage from nil to 50%, whereas oxidative processes and photodegradation present almost total removal, but toxic by-products can be produced. For these reasons, the adsorption process has become a popular method for the removal of organic contaminants (i.e. PPCPs, petroleum derivates and dyes) from aqueous matrices. The strength of adsorption processes is their inexpensiveness and simplicity^14^. Activated carbons have largely been used as adsorbent material due to their hydrophobicity, surface functionality, pore structure and high surface area^15,16^. Other carbon-based materials, such as multi-walled carbon nanotubes^17,18^ and graphene/graphite^19,20^ compounds, have already been used as adsorbent material, showing high efficiency in removing (closed to 100%) PPCPs from water.

Diclofenac sodium (DCF) is an analgesic drug included in the class of PPCPs, generally prescribed to treat inflammatory disorders because of its nonsteroidal anti-inflammatory potential. The DCF neutral form presents free acid groups (–COOH), whereas the anionic form presents deprotonated acid groups (–COO^−^). Chronic exposure to DCF generates hemodynamic changes and thyroid tumors in Humans^2^. Furthermore, negative effects have been observed on natural ecosystems, causing the death of several animal species. Particularly, researchers investigated on the death of a popular specie of vultures in South Asia, proving that eating carcasses of animals nursed with DCF may cause visceral gout in vultures, mainly due to the crystallization of their internal organs^21^. DCF has largely been detected in both natural water and wastewater worldwide because of its common use in large quantities^22^. Therefore, its removal by using adsorption process has attracted the attention of the research community. In this study, the thermo-plasma expanded graphite (TPEG) was used to remove DFC from aqueous solutions. Expanded graphite consists of laminated two-dimensional nanoparticles bound to each other by van der Waals forces only, and it is produced by the expansion of natural graphite. This process confers excellent physic-chemical properties to TPEG, for example an apparent density^23^ in the range 2.3 to 9 g L^−1^, that makes it an excellent adsorbent material. Furthermore, it has a typical fibrous morphology and overlapping of graphene layers can be evidenced by SEM analysis. Packing of more than five graphene layers are the basic structure of TPEG with presence of mesopores and micropores. TPEG was used as an adsorbent material and its interaction with DCF was investigated. TPEG was chosen as adsorbent material because it is produced by an industrial process that ensures a significative increase of surface area of the starting material promising good potential adsorbent properties. The industrial process of the production is covered by industrial secret but basic information are available and it is known that the process involves a first step of chemical intercalation of natural graphite and a second step of very fast expansion conducted by a plasma thermal heating. That ensures an expansion bigger than the typical expansion process. Due to its industrial production that ensures a not very expensive cost, promising improvement of mechanical characteristic, increase of surface area and changing in the apparent density, TPEG resulted to be an interesting material to investigate for environmental remediation purpose as adsorbent material. All that represents the novelty of the material investigated and its application could be a significant progress into the field of the water treatment and remediation. Furthermore, its use to the removal of an actual ubiquitous pollutants that is not well removed by traditional wastewater plants represents a further point of interest of the reported work. Therefore, experimental studies about the mechanisms of interaction between TPEG and DCF, the kinetics and the thermodynamics of the process, pH influence and the reuse of the adsorbent material, were conducted. Expanded graphite was already investigated as adsorbent material for several pollutants and it was used as pure^24,25^ and composite^26,27^ form. That encourages that work and the aim to investigate the use of this innovative form of expanded graphite to remove new kind of pollutants and expect significative adsorption capacity.

## Materials and methods

The experimental setup used to investigate the adsorption mechanism of the adsorption of DCF on the TPEG was inspired to previous work reported in literature^28–30^ focused on removal of pollutants from water by adsorption. For this reason, the procedure reported from the Sections “Experimental setup” to “Thermodynamics of the process” contains some description used by the previous cited work.

### Materials

TPEG was obtained from Innograf (Potenza, Italy). The initial pH of the solutions was adjusted by adding NaOH and HCl purchased from Carlo Erba reagents (Carlo Erba, Rodano, Milano, Italy) and Diclofenac sodium salt was supplied from Sigma Aldrich (Sigma-Aldrich, Schnelldorf, Germany, purity: 99.9%). The stock solution of DCF was prepared in distillated water at a concentration of 100 mg L^−1^. All reagents were of extra pure grade and used without further purification.

TPEG is obtained from natural graphite by means of chemical intercalation followed by the thermal plasma expansion at high temperatures. Different methods, such as chemical vapor deposition and chemical intercalation, are available to expand the natural graphite. TPEG used in this study is produced by means of an innovative process consisting in the chemical intercalation of natural graphite followed by high temperature thermal plasma expansion. This process separates graphite in different layers, with a volume expansion of up to 300 units, compared to an average of 200 units obtainable by other standard methods. TPEG has good structural properties, such as mechanical strength of about 1 TPa, a thermal conductivity of about 500 W mK^−1^ and a diameter between 60 and 300 µm^31^.

### Material characterization

SEM (scanning electron microscope) images were obtained by using a high-resolution field emission scanning electronic microscopy (HR-FESEM), Auriga Zeiss model, at CNIS laboratory of the University of La Sapienza (Rome, Italy).

TEM images was obtained by using transmission scanning electronic microscopy (TEM), FEI-TECNAI G2 20 TWIN model, with a value of 120 kV of acceleration voltage.

XRD (X-ray powder diffraction) spectra have been acquired by a X-Perth-Pro Philips X-ray diffractometer, operating at 40 kV and 32 mA, using CuKα radiation (wavelength of 1.5406 Å) in a q-2q configuration. The spectra have been acquired at 2q 10°–80°, step size 0.040°, time per step 4 s. The Scherrer equation was used to calculate the average dimension of crystallite (stacking of graphene’s sheet), while the average number of sheets of each stacking was obtained by dividing the dimension of crystallite for the reticular distance obtained by using the Bragg equation. Scherrer and Bragg equation are reported (Eqs. 1 and 2). τ is the main size of the ordered crystalline domains, K is the shape factor, λ is the X-ray wavelength, β is the line broadening at half maximum intensity, θ is the Bragg angle, n is a positive integer and d is the interplanar distance.

1 $$ \tau = \frac{K\lambda }{{\beta \cos \theta }}\;\;\;\;\;\;\;\;\left( {{\text{Scherrer }}\;{\text{equation}}} \right) $$

2 $$ n\lambda = 2d\sin \theta \;\;\;\;\;\;\;\;\; \left( {{\text{Bragg }}\;{\text{equation}}} \right) $$

Micro-Raman analysis was carried out by using a Jobin–Yvon Horiba LabRam microRaman-spectrometer, equipped with a He–Ne laser (λ = 632.8 nm), an edge filter and an Olympus microscope with 10 ×*/*50 ×*/*100 × objectives. A spectral resolution of about 5 cm^−1^ was obtained by a holographic grating with 600 grooves mm^−1^. Spectra were acquired with an accumulation time of 60 s and a laser power of 20mW.

Specific surface area was measured with MONOSORB quantachrome instrument by applying the BET (Brunauer–Emmett–Teller) single point technique and by using N_2_/He 30% as gas to adsorb/desorb and BET multipoint technique by using ASAP 2020 instrument of Micromeritics.

The pH of zero charge was also evaluated by adding different amount of GTPEG in a solution with different initial pH and evaluating the final pH after 24 h of contact. The ΔpH observed for the different initial pH was reported in a graph and the pH_ZPC_ was identified as the point with a value of ΔpH of 0.

FT-IR spectrum was obtained in the range 400–4000 cm^−1^ (16 cm^−1^ of resolution) by using a ThermoNicolet 5700 FT-IR spectrophotometer (Thermo Fischer Scientific, https://www.thermofisher.com/be/en/home.html). The sample was measured in the form of KBr pellet, prepared by mixing 0.2 g of sample to 20 g of KBr (stored in the oven at 105 °C to eliminate trace of humidity), crushedby hand in a mortar and pressed at 9 tons cm^−2^. The characterization of material was done consistent with literature information^32,33^.

### Experimental setup

In order to investigate the influence of pH on the adsorption process, experimental batch adsorption tests were performed by adding 10 mg of TPEG to 50 mL of DCF solution (100 mg L^−1^) in a conical flask. The initial pH was adjusted at different values (i.e., 1, 2, 3, 5 and 7) by adding NaOH and HCl and monitored by an Orion 420A pH meter (ThermoFisher Scientific, Waltham, Massachusetts, USA). The conical flask was placed on a magnetic stirrer (IKA RH digital) and was mixed at 650 rates per minute (rpm) at room temperature for 22 h to ensure contact between TPEG and the contaminated matrix. After treatment, the supernatant was aspirated to remove TPEG and the amount of residual DFC was measured. Therefore, the adsorption capacity was evaluated at different pH values, as follows:

3 $$ q = \frac{{mass\; of\; DCF\; adsorbed \left( {mg} \right)}}{mass\; of \;adsorbent \left( g \right)} $$

4 $$ q = \left( {{\text{c}}_{{\text{i}}} - {\text{ c}}_{{\text{f}}} } \right){ }\frac{{\text{V}}}{{\text{m}}} $$

where ${\mathrm{c}}_{\mathrm{i}}$ and ${\mathrm{c}}_{\mathrm{f}}$ (mg L^−1^) are the DFC concentrations at the beginning and after each adsorption experiment, V is the initial solution volume (L), and m is the adsorbent weight (g).

Removal was also evaluated by the ratio between mass of adsorbed DCF and initial DCF present in the solution.

### Analytical methods

DCF residual concentrations were estimated by UV–Vis spectrometry at 271 nm (Dr. Lange Cadas 200 spectrophotometer) with the calibration line method. The absorbance of DCF solution was measured at pH 3.3

All the tests were repeated three times to obtain the mean value.

### Initial concentration influence

The influence of initial concentration of the pollutant on the adsorption capacity of TPEG was also evaluated. 50 mL of DCF’s solution at different initial concentration (250, 200, 100, 40, 20, 10 mg L^−1^) at pH 1 was mixed with 10 mg of TPEG and stirred at 650 rpm for 10 min. Then, the residual concentration of DCF was evaluated and the adsorption capacity calculated. Each test was repeated three times.

### Adsorption kinetics models

The kinetics models investigate the velocity of the process, providing the relationship between contact time and adsorption capacity. In order to study the kinetic of the process, experimental tests were executed by mixing 10 mg of TPEG with a 50 mL sample of contaminated water with a DFC concentration of 100 mg L^−1^, at 650 rpm as the stirring speed and 1 as the initial pH. Using different contact times, i.e. 3, 5, 7, 10, 15, 20, 30 and 40 min, the adsorption capacity was determined. The obtained data were plotted to evaluate the correlation between contact time and adsorption capacity. In order to evaluate the real mechanism of DFC adsorption on TPEG, the experimental data were fitted to five kinetic mathematical models, i.e. pseudo-first order, pseudo-second order, intraparticle diffusion, Elovich and liquid film diffusion models. The best fitting was identified through the calculation of the R^2^ coefficient. Table 1 shows the linear form of the model equations. All tests were replicated three times to obtain the mean value. The kinetics model used in this paper, are pseudo-first order model^34^ (Eq. 5), pseudo-second order model^34^ (Eq. 6), Elovich model^35^ (Eq. 7), liquid film diffusion model^36^ (Eq. 8) and intraparticle diffusion model^36^ (Eq. 9).

**Table 1:** Fitting of experimental data with theoretical kinetics models.

Model	R^2^	Parameters
Pseudo-first order	0.5519	K_1_ = 3.44·10^–2^ min^−1^ q_e_ = 10^5^ mg g^−1^
Pseudo-second order	0.9818	q_e_ = 400 mg g^−1^ k_2_ = 6.6·10^–4^ g mg^−1^ min^−1^
Elovich	0.7222	β = 1.2·10^–2^ mg g^−1^ min^−1^ α = 267.5 g mg^−1^
Liquid film diffusion	0.5519	K_fd_ = 0.0334 min^−1^
Intraparticle diffusion	0.5873	K_dif_ = 40.812 mg g^−1^ min^-1/2^ C = 143.43

Fitting of different kinetic models for adsorption of DCF on TPEG.

5 $$\mathrm{log}\left({q}_{e}-{q}_{t}\right)=\mathrm{log}\left({q}_{e}\right)-{k}_{1}t$$

6 $$\frac{t}{{q}_{t}}= \frac{1}{{k}_{2}{q}_{e}^{2}}+\frac{t}{{q}_{e}}$$

7 $${q}_{t}=\frac{1}{\beta }\mathrm{ln}\left(\alpha \beta \right)+\frac{1}{\beta }\mathrm{ln}\left(t\right)$$

8 $$\mathrm{ln}\left(1-\frac{{q}_{t}}{{q}_{e}}\right)=-{k}_{fd}t$$

9 $${q}_{t}={k}_{dif}{t}^{1/2}+C$$

where q_e_ is the equilibrium adsorption capacity (mg g^−1^), q_t_ is the adsorption capacity at time t (mg g^−1^), k_1_ is the rate constant of pseudo-first order(min^−1^), k_2_ is the rate constant of pseudo-second order (g mg^−1^ min^−1^), α and β are the initial adsorption rate of the Elovich Equation and the desorption constant related to the extent of surface coverage and activation energy constant for chemisorption (mg g^−1^ min^−1^) (g mg^−1^), k_fd_ is the liquid film rate diffusion constant (min^−1^) and k_dif_ are the rate constant of intraparticle diffusion (mg g^−1^ min^−1/2^).

### Adsorption isotherms

The analysis of the adsorption isotherms is important to identify the interaction between the residual concentration of pollutant in water samples and the adsorption capacity of the adsorbent. Therefore, adsorption capacity was evaluated for different DCF concentrations (i.e. 10, 20, 40, 100, 200 and 250 mg L^−1^). 10 mg of adsorbent were mixed with 50 mL of DCF solution, stirred for 10 min at 650 rpm at pH 1 (optimal pH) and the obtained experimental data were fitted using Langmuir^37^, Freundlich^38,39^, Temkin^40^ and Dubinin–Radushkevich^41^ isotherm models. All the tests were repeated in triplicate. In order to evaluate the best fit of the isotherms to the experimental data, the coefficient of linear regression (R^2^) was calculated.

The Langmuir model is valid for monolayer adsorption on a surface containing a finite number of identical sites and the adsorption is uniform. The linear form of the Langmuir equation is:

10 $$ \frac{{C_{e} }}{{q_{e } }} = \frac{1}{{K_{L } q_{m} }} + C_{e } /q_{m} $$

where C_e_ is the concentration of adsorbate at equilibrium in the liquid phase (mg L^−1^), q_e_ is the adsorption capacity at equilibrium (mg g^−1^), K_L_ (L mg^−1^) is associated to free energy of the process and q_m_ (mg g^−1^) is the maximum adsorption capacity (mg g^−1^).

The Freundlich model assumes a multilayer adsorption and the surface of adsorbent contains a set of nearby sites. The linear form of the equation is:

11 $$ \ln q_{e} = \ln K_{f} + \frac{1}{{n_{F} }}lnC_{e} $$

where K_f_ roughly indicates the adsorption capacity [(mg g^−1^) (L mg^−1^)^1/n^] and $\frac{1}{{n}_{F}}$ defines the adsorption intensity.

The Temkin model assumes the linear decrease of adsorption heat of all molecules in the layer with the coverage due to the adsorbent–adsorbate interaction. Moreover, it supposes that the adsorption is characterized by a uniform distribution of the binding energies, up to some maximum binding energy. The linear form of the equation is:

12 $${q}_{e}= {B}_{1}\mathrm{ln}A+ {B}_{1}\mathrm{ln}{C}_{e}$$

where A is the equilibrium binding constant (L g^−1^) and B_1_ is related to the heat of adsorption (J mol^−1^).

The Dubinin–Radushkevich model assumes that the adsorption occurs on a heterogeneous surface with a steric hindrance between adsorbed and incoming particles. The linear form of the equation is:

13 $$\mathrm{ln}\left({q}_{e}\right)=\mathrm{ln}\left({q}_{s}\right)-\beta {\varepsilon }^{2}$$

14 $$\varepsilon =RTln(1+\frac{1}{{C}_{e}})$$

15 $$E= \frac{1}{-\sqrt{2\beta }}$$

where E is related to free energy (kJ mol^−1^), β is the Dubinin–Radushkevich constant (mol^2^ J^−2^), q_s_ is the adsorption capacity (mg g^−1^) and ε is the Polanyi potential.

### Thermodynamics of the process

The thermodynamic study allows an understanding of the relationship between spontaneity, free energy, and temperature during the process. Moreover, it gives information about the temperature at which the process is at equilibrium under standard conditions.

In order to evaluate the thermodynamics of the process, the adsorption capacity was determined for different temperature values (i.e. 293, 299 and 311 K) by adding 10 mg of adsorbent to 50 ml of DCF solution (100 mg L^−1^). The conical flask was placed on a magnetic stirrer and was mixed at 650 rpm for 10 min. Each test was repeated in triplicate. The following relationships allowed us to evaluate the thermodynamics of the process^42^:

16 $$ \Delta G^{0} = - RT\ln \frac{{q_{e} }}{{C_{e} }} $$

17 $$ \Delta {\text{G}}^{0} = \, \Delta {\text{H}}^{0} - {\text{ T}}\Delta {\text{S}}^{0} $$

where ΔG^0^ is the standard free energy, q_e_ is the adsorption capacity, C_e_ is the concentration of adsorbate at equilibrium, ΔH^0^ is the standard enthalpy, T is the temperature and ΔS^0^ is the standard entropy.

From Eqs. (16) and (17), the following equation can be obtained:

18 $$ ln\frac{{q_{e} }}{{C_{e} }} = - \, \Delta {\text{H}}^{0} /{\text{RT }} + \, \Delta {\text{S}}^{0} /{\text{R}} $$

Therefore, in order to understand the driving force of the process, enthalpy and entropy of the process were evaluated by plotting $ln\frac{{q}_{e}}{{C}_{e}}$ versus 1/T.

### Regeneration and reuse of TPEG

The possible reuse of TPEG after a regeneration process is important to obtain subsequent technical and economic advantages. Two different regeneration processes were tested to recover the material for subsequent remediation tests. Solvent washing was carried out in a glass beaker by adding exhausted TPEG to 50 mL of NaOH (0.2 M). The solution was posed on a magnetic stirrer and mixed at 800 rpm for 3 h.

The thermo treatment was performed by regenerating TPEG samples in an oven at firstly 105 °C for 2 h and then at 200 °C for 4 h. Before the treatment, the material was separated from DCF solution by aspirating the liquid phase with a pipette.

The regenerated TPEG was reused to perform 2 and 4 cycles of treatment after solvent washing and thermo-recovery, respectively. Particularly, 10 mg of TPEG were added to 50 mL of DCF solution (20 mg L^−1^, pH 1) and mixed at 650 rpm for 10 min on a magnetic stirrer. Each test was repeated in triplicate.

The performance of regenerated TPEG was evaluated by comparing the adsorption capacity using pure and regenerated material, as following (Eq. 19):

19 $$Relative \,{q}_{e}= \frac{DCF \, adsorbed \, at \, specific \, cycle (mg)}{DCF \, adsorbed \, at \, first \,  use (mg)} \times 100$$

## Results and discussion

### Material characterization

**Figure 1:**
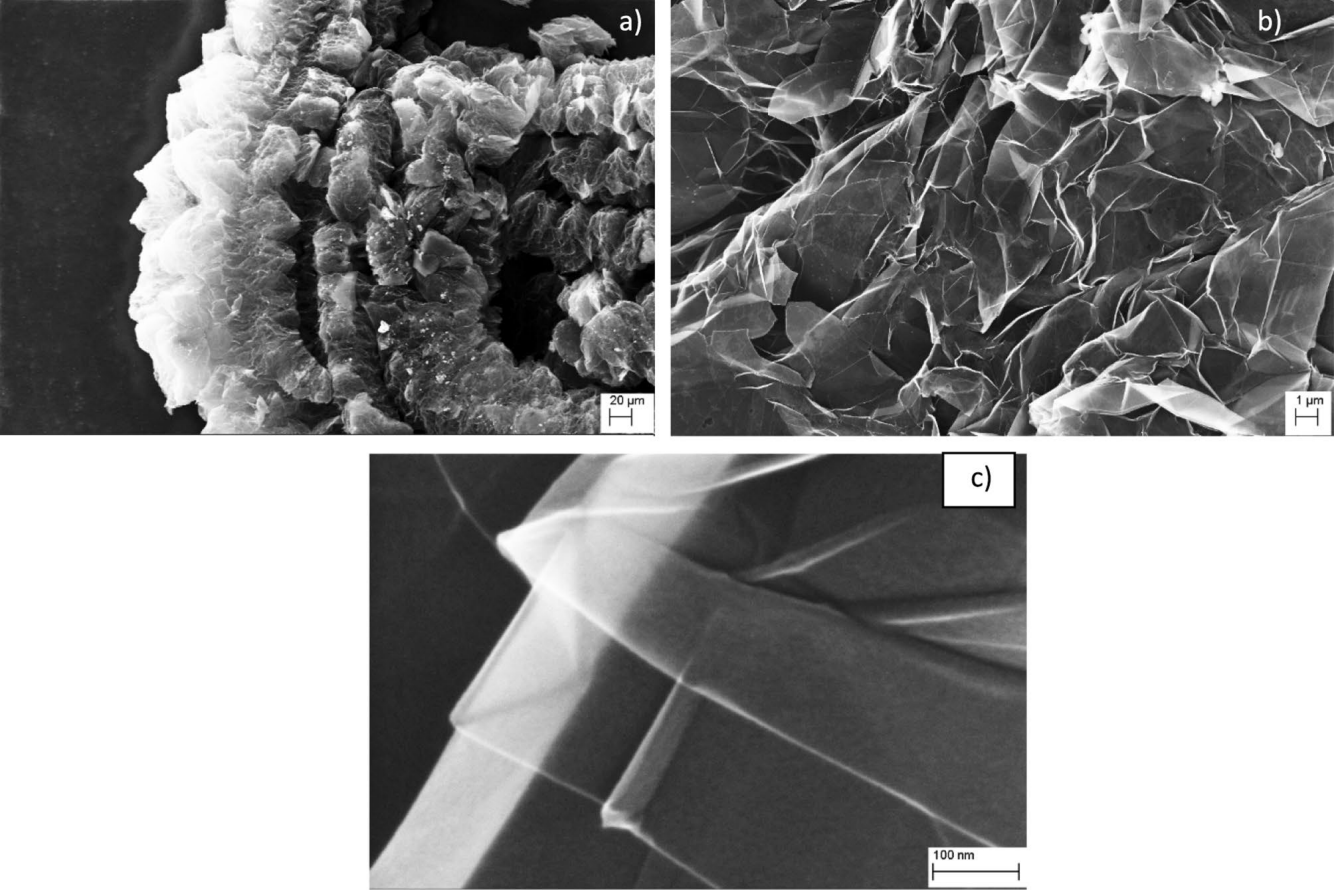
SEM images of the TPEG with different magnification (**a**: 500 ×, **b**: 10,000 ×, **c**: 390,400 ×).

**Figure 2:**
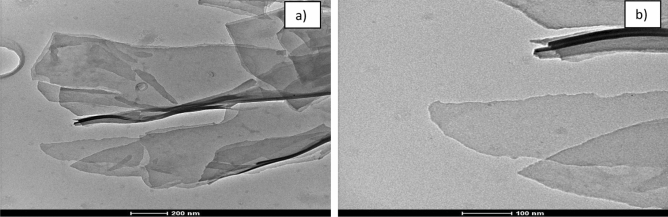
TEM images of TPEG. The different scales of the images are reported to evidence the different magnifications.

In the Figs. 1 and 2, the SEM and TEM images with different magnification of the material are reported.

**Figure 3:**
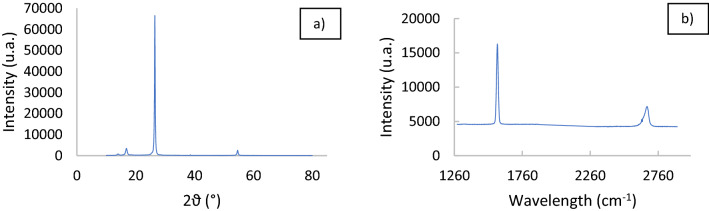
(**a**) difractogramm of TPEG, (**b**) Raman spectrum of TPEG.

By observing SEM images of the material, it is clear the presence of different sheet of graphene packaged together to form a series of layers of sheet of graphene that form a fibrous macrostructure. It is possible to observe a crumple-like structure which is common in graphene. TEM images confirm the presence of multilayer of graphene^43,44^. Thin stacked of various size and shape with a multilayered structure are present. X-ray diffractogram and Raman spectrum obtained are reported in Fig. 3.

X-ray diffraction and Raman analysis confirmed the packing of different sheet of graphene (number of layers > 5), because the typical diffractogram of graphite (position and broad of peaks) and a typical Raman spectrum of graphite or sheet of graphene packed (n > 5) were obtained^45^ (ratio of the intensity of the peak I_D_/I_G_ of about ½, where I_D_ is the peak at about 2679 cm^−1^ and the I_G_ is the peak at about 1577 cm^−1^). From XRD and Raman analysis results, it is possible to conclude that the layers of graphene stacked are minimum 5^45^.The typical peaks of graphite at about 26.5° and 55° were obtained and the dimension of crystallites (stacking of graphite’s sheets) and d-spacing were calculated from the shape and position of the peak at 26.5° by using the Scherrer and Bragg equation. The dimension of 24.02 nm was calculated as crystallite dimension, while 0.374 nm was obtained as d-spacing. The relative intensity of the peak, G and D, present in Raman spectrum (about 1577 and 2679 cm^−1^) obtained and the position and the shape of the peak D are typical for a graphitic system or packed graphene’s sheet (n > 5) that has a broad D peak. Therefore, the material presents a series of sheets (n > 5) of graphene packed to form a ordered structure. The BET analysis reveals an important information about the structure of material, mesopores and micropores are present and the specif surface area of the mesopores is about 18 m^2^ g^−1^, while the total surface area of the TPEG is about 45 m^2^ g^−1^. Therefore, the surface area of micropores is 27 m^2^ g^−1^. The value of the surface area, as reported in detail in the Section “Adsorption isotherms” for the comparison of the performance of the material, resulted to be into the large range of the BET surface observed for adsorbent materials tested for the DCF removal by the adsorpation.

**Figure 4:**
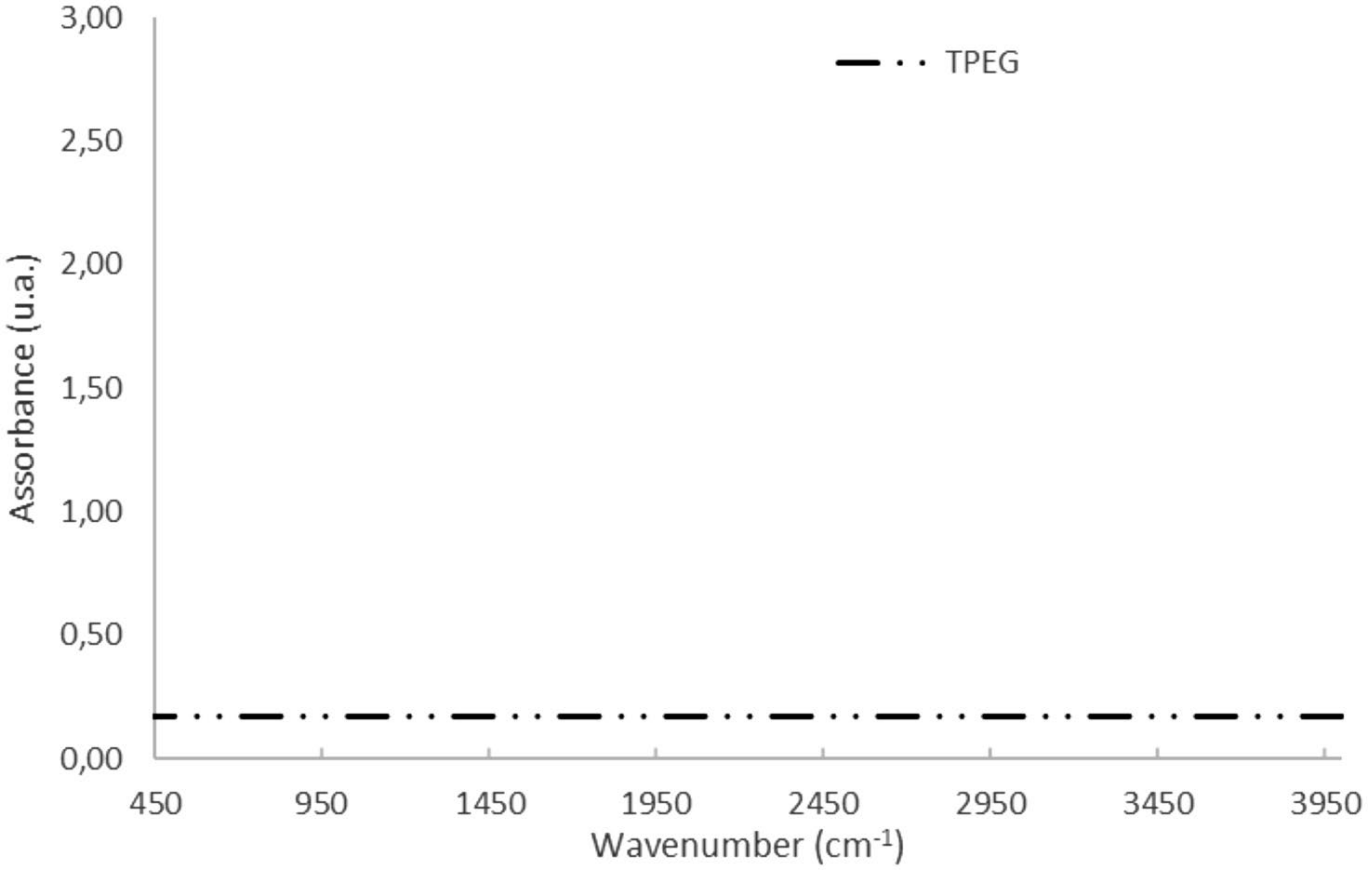
FT-IR spectrum acquired in the range 400–4000 cm^−1^ and resolution of 16 cm^−1^.

In the Fig. 4 the FT-IR of the TPEG are reported. No peak of functional groups were detected and it confirms that the TPEG has a typical structure of the graphitic pure substances. The thermo-plasma expansion conducted into inert chamber ensures no oxidation of the graphite.

### pH influence on the adsorption capacity

**Figure 5:**
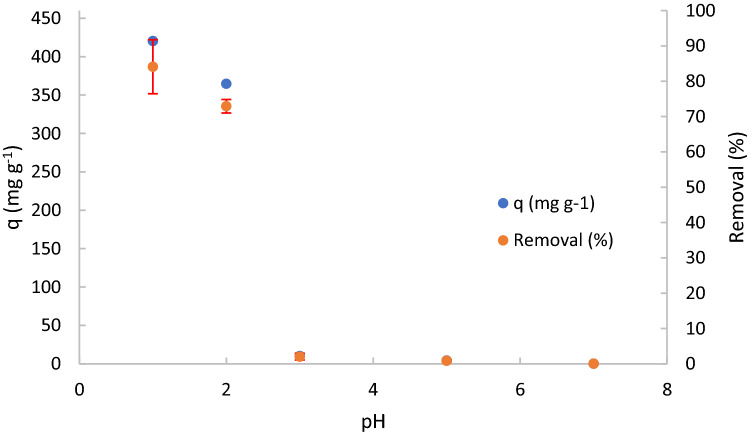
Effect of pH on DFC removal by TPEG and its adsorption capacity. C_o_ = 100 mg L^−1^, contact time = 22 h, Solution volume = 50 mL, mass of adsorbent = 10 mg, stirring speed = 650 rpm. Standard deviation is also reported for each experimental data.

Figure 5 shows the variation of TPEG adsorption capacity and removal by varying the pH in the range 1–7. Some value of removal and adsorption capacity are overlapped but this scale of removal is used in agreed with the next figures.

**Figure 6:**
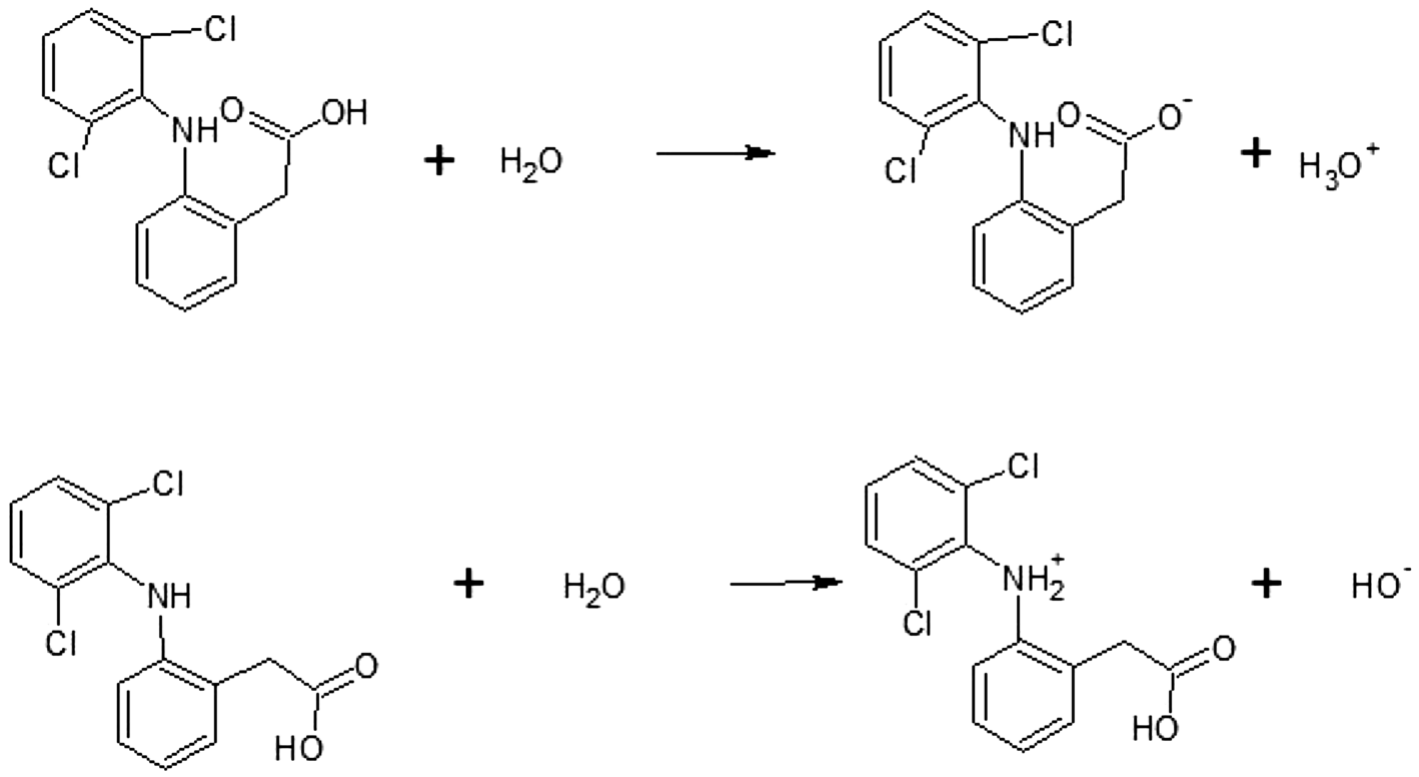
Equilibrium dissociation of DCF.

Adsorption capacity is maximum (420.5 mg g^−1^) at pH 1 and 2, i.e. at values higher than pKa. Adsorption capacity decreases with increasing pH, reaching the minimum (closed to 0) at pH 3, i.e. at values close to pKa. The reason for this is probably that DFC solubility decreases^46^ at low pH values due to the presence of DCF in the neutral form, resulting in a better adsorption capacity. Therefore, the optimal value of pH for this study is 1. The solubility of DCF in the pH range of 1.0 to 4.5, is almost between 1.2 to 3.6 mg L^−1^. That values are lower than the value at higher pH range^46^. The Fig. 6 illustrates the dissociation equilibrium of DCF to better understand the influence of pH on its grade of dissociation and therefore on its solubility.

As can be observed, DCF has a carboxylic acid group able to react with water (acid–base reaction) to form the conjugate anion of DCF and hydroxonium. Because of DCF, as all organic acid, is a weak acid, the reaction of dissociation is an equilibrium dissociation and it is influenced by the external presence of H_3_O^+^ or OH^-^. In detail, when the pH is acid ([H_3_O^+^] > 10^–7^ M) the equilibrium moves to reagents of the reaction reported in the Fig. 4 because the presence of H_3_O^+^, therefore DCF is present in solution in its undissociated form. When the pH is basic ([OH^-^] > 10^–7^ M) the equilibrium moves to the product of the reaction because H_3_O^+^ reacts with OH^-^ present in the solution, therefore DCF is largely present in its dissociated form. The amino group of DCF can also react with water, as reported in the Fig. 4, to form the conjugate cation of DCF and hydroxide ion. The pH has the same reverse effect described for the carboxylic group. Therefore, the form of DCF in the solution depends on the pH of the solution and it is neutral for pH values less than its pKa (4.2)^33,47^. The pH_ZPC_ of the TPEG was 12, therefore in the range of the pH considered, the charge of the TPEG was positively and electrostatic interaction between undissociated/dissociated form of DCF and TPEG is not affected by the change of the TPEG charge surface. It confirms that the increase of adsorption capacity by decreasing the pH in mainly related to the solubility of DCF decrease, for the value upper than 4.2 the increase of negatively charge of DCF does not compensate the increase of solubility and no further increase of adsorption capacity was observed.

By considering the results observed, π-π interactions can be excluded because the number of DCF^33^ and TPEG π-electrons do not change by changing the pH. Therefore, adsorption capacity is not influenced by pH in the case of these interactions as principal adsorption mechanisms.

### Initial concentration influence

**Figure 7:**
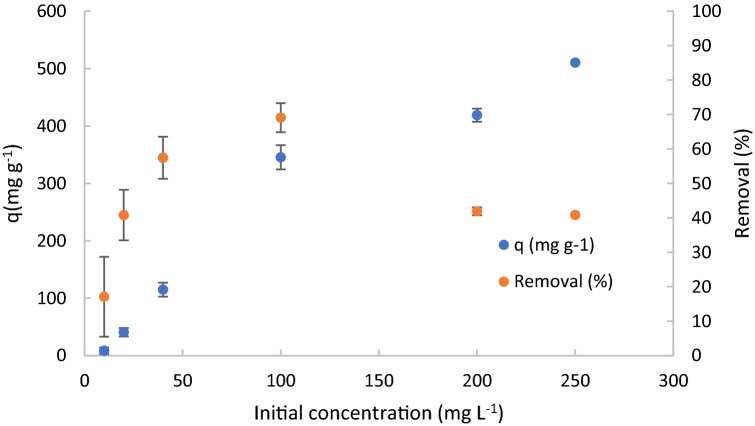
Adsorption capacity and removal for different Initial concentration of DCF. C_0_ = 10, 20, 40, 100, 200 and 250 mg L^−1^, pH 1, 10 mg of adsorbent material, stirring speed = 650 rpm, contact time = 10 min.

The Fig. 7 shows the influence of initial concentration of DCF on adsorption capacity and removal. Their standard deviations are also reported.

A rapid increase of adsorption capacity was observed at the initial increase of initial concentration of DCF, followed by a slow increase of adsorption capacity for initial concentration higher than 100 mg L^−1^ probably caused by the saturation of the material. The observed behavior is the typical behavior observed in adsorption processes caused by the occupation of the TPEG adsorption sites involved into the interaction with adsorbate (DCF). When all the adsorption sites are occupied no further adsorption can be observed and the increase of concentration of adsorbate cannot involve other interaction with adsorbent. When that equilibrium is reached, increase of dosage of adsorbent is necessary to have further adsorption. The same behavior was observed for the removal, but for the initial concentration higher than 100 mg L^−1^ a decrease of removal was observed because the increase of concentration are not proportional to the increase of number of active sites occupied by the adsorbate (DCF). The values of 200 was evidenced as the ratio [DCF]/[TPEG] of maximum removal observed, therefore for values of initial concentration of DCF higher than 100 mg L^−1^ an increase of TPEG are necessary to maintain the ratio at 200 and ensure the maximum removal reached (69%). For that described reason, in the scale up of the process it is necessary to decide what parameters would be maximized for value of initial concentration higher than 100 mg L^−1^. In order to maximize the removal, more than 10 mg of TPEG are required but a decrease of adsorption capacity will be observed because of more adsorbent material will be used.

### Adsorption kinetics

**Figure 8:**
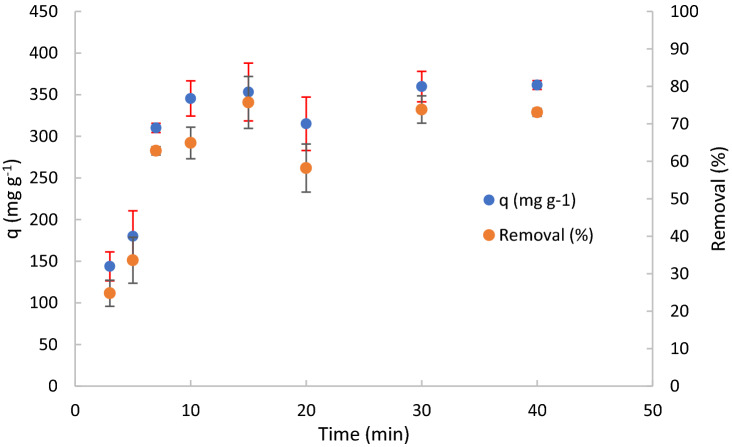
Kinetic trend and standard deviation of experimental data of adsorption capacity and removal with contact time. C_o_ = 100 mg L^−1^, Solution volume = 50 mL, pH 1, mass of adsorbent = 10 mg, stirring speed = 650 rpm. Some values of standard deviation are lower than surface area of indicators.

Figure 8 shows the study of kinetics adsorption by varying the contact time in the range 3–40 min. Removal and adsorption capacity observed at different time of contact are reported.

An increase of adsorption capacity with the increase of contact time can be observed in the first 10 min of treatment, then a plateau is reached. Therefore, the optimum contact time is 10 min, corresponding to the maximum adsorption capacity of about 350 mg g^−1^. Table 1 summarizes the kinetic parameters and the coefficient of correlation obtained by fitting experimental data with the previous cited kinetic models.

The results show that the pseudo-second order model is the best fitting of the experimental data. Particularly, the adsorption capacity obtained from the pseudo-second order kinetic model (400 mg g^−1^) is close to the experimental data obtained for long contact time (i.e. 22 h, 420.5 mg g^−1^). The value of kinetic constant (k_2_:6.6·10^–4^ g mg^−1^ min^−1^, at room temperature) is comparable with that obtained in literature (i.e. 10^–2^/10^–3^)^33,47^. Because of the pseudo second order model regulates the process, it is possible to conclude that by increasing two times the concentration of DCF an increase of four times of the rate of the process are observed. By considering that the kinetics model that regulate the process is the pseudo-second order model, we can conclude that the slowly step of the process resulted to be the interaction (the formation of the bond) between adsorbate and adsorbent active sites. By the pseudo-second order model is not possible to distinguish between physical adsorption, such as electrostatic interaction, and chemical adsorption, such as amide formation. To evaluate about the type of the interaction, thermodynamics evaluation, by considering the free energy, is necessary and for this reason were conducted and reported in Section “Thermodynamic study”. To resume here the results obtained, physical-adsorption can be designed as mechanism of adsorption of DCF on TPEG because of the free energy value lower of 30 kJ mol^−1^. Van der Waals force and other hydrophobic interactions can be designed as main interactions involved into the adsorption process. As expected, liquid film diffusion rate step was increased by the agitation of the solution and it was not the limiting step of the process. The intraparticle diffusion step was not the limiting step probably due the dimension of pores that ensures fast diffusion of the molecules of DCF into the pores and reaching and approaching the active surface of the adsorbent (dimension of pores >> dimension of DCF). Elovich and pseudo-first order models also involve as limiting step the interaction between adsorbate and adsorbent active sites but they differ from pseudo-second order model because the adsorbent site are heterogeneous for Elovich model and the rate of the process increase two times if an increase of two times of adsorbate (DCF) is observed. By considering that, to improve the rate of the process modification of the surface of the material must been conducted. Amino functionalization of the surface could improve the reactivity of the material for the adsorption of DCF, because the presence of the amino group could interact with acid group of DCF and form amide bond. If amino functionalization will be conducted, the addition of substance in the solution or adsorbed on TPEG surface with the capacity of catalyze the amide bond could further improve the rate of the process. By considering the kinetics studies, it is possible to conclude that diffusion of DCF into the mesopores and micropores of TPEG to approach the surface of it, where are involved physical interactions able to “entrap” the molecules of DCF. The same shape of the adsorption capacity was observed obviously for the removal. By increasing the time of contact an increase of removal until to reach the maximum removal of about 73%. The maximum removal was almost reached after ten minutes of contact.

### Adsorption isotherms

**Figure 9:**
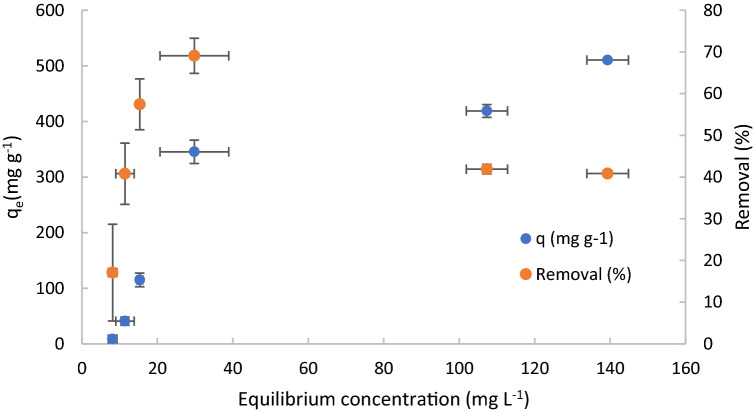
Equilibrium adsorption of DCF on TPEG. C_0_ = 10, 20, 40, 100, 200 and 250 mg L^−1^, pH 1, 10 mg of adsorbent material, stirring speed = 650 rpm, contact time = 10 min. Standard deviation for adsorption capacity and equilibrium concentration are also reported (some values are lower than the indicator surface).

Figure 9 shows the obtained results for the removal and adsorption capacity for different value of equilibrium concentration of DCF in the solution.

As expected, the adsorption capacity increases with the increase of the pollutant concentration, reaching the adsorbent saturation represented by the plateau. The removal of DCF reach a maximum value and then it decreased because the increase of molecules of DCF is not proportional to the increase of the number of active sites occupied.

The results of experimentations demonstrate that the best fitting was obtained with the Dubinin–Radushkevich model with R^2^ = 0.9903, proving that the adsorption occurs on a heterogeneous surface with steric hindrance between adsorbed and incoming particles. For the Langmuir model, Freundlich model and Temkin model values of R^2^ of 0.058, 0.7596 and 0.9372 were obtained, respectively. Values of 433.29 mg g^−1^, 5·10^–5^ mol^2^ J^2^ and − 0.1 kJ mol^−1^ were observed for adsorption capacity, constant of Dubinin–Radushkevich and free energy, respectively, calculated from the model. Particularly, the value of adsorption capacity calculated from this model (433.29 mg g^−1^) is in good accordance with the value calculated from kinetic study (400 mg g^−1^). The Dubinin–Radushkevich model are typical of process of adsorption that involve a formation of multilayers. Normally, multilayer adsorption involves two or more plateau of adsorption capacity because of a multilayer of adsorbate can be formed after the totally covering of adsorbent surface. In the Fig. 9, just one plateau can be observed, probably because the first one could be observed for equilibrium concentration lower than that investigated in this work. Furthermore, to prevent the steric hindrance between adsorbed molecules and incoming ones, can be useful to increase the surface area of TPEG to delay that hindrance as much as possible and increase the adsorption capacity and formation of more layer of adsorbate. Furthermore, the adsorption isotherm shape seems to have the typical shape of adsorption isotherm of type IV, characteristic of system that present mesopores and phenomena of pore blocking caused by the steric hindrance. Another consideration useful that can be deducted by the isothermal fitting is the best correlation with model that assume heterogeneous surface of the adsorbent material (Temkin, Dubinin–Radushkevich). In the Table 2 are reported the parameters obtained from the isothermal models investigated.

**Table 2:** Fitting of experimental data with theoretical isothermal models.

Model	Parameter	
Langmuir	R^2^	0.058
	q_m_	769 mg g^−1^
	k_L_	0.003 L mg^−1^
Freundlich	R^2^	0.7596
	n_F_	0.85
	K_F_	6.39 (mg g^−1^) (L mg^−1^)^1/nF^
Temkin	R^2^	0.9372
	B_1_	173 J mol^−1^
	A_1_	0.01 L g^−1^
Dubinin–Radushkevich	q	0.9903
	R^2^	433.29 mg g^−1^
	β	5 × 10^−5^ mol^2^ J^−2^

Fitting of different isothermal models for adsorption of DCF on TPEG.

The comparison between our results and those obtained in literature using other adsorbent materials is reported in Table 3, demonstrating the excellent adsorbent properties of TPEG.

**Table 3:** Comparative data of adsorbent material for removal of DCF.

Adsorbent material	Adsorption capacity (mg g^−1^)	Interaction	Reference
Thermo-plasma expanded graphite	433.29 (from isotherm) 400 (from kinetics)	Hydrophobic and heterogeneous carbon surface	This work
Grape bagasse	77	Electrostatic	^28^
CNT/HNO_3_	24	–	^17^
CNT/Al_2_O_3_	27	π-π and van der Waals force	^18^
Expanded graphite	330	Hydrophobicity and energetically uniform carbon surface	^48^
GO	500	Hydrophobic and π-π	^48^
UiO-66	189	Electrostatic	^49^
OAC (2.0)	487	Electrostatic and H-bonding	^33^
Commercial AC	76	Electrostatic	^33^
Zeolite modified with cetylpyridiumchloride	160	Electrostatic and hydrophobic	^50^

Comparison on adsorption capacity of DCF on different adsorbent material.

In some cases, the higher adsorption capacity observed cannot be related to the surface area of the material (47 m^2^ g^−1^) because it is not higher than the values observed for some materials listed in the table for the comparison, such as CNT/Al_2_O_3_ (237 m^2^ g^−1^), UiO-66 (1710 m^2^ g^−1^) and zeolite modified with cetylpyridiumchloride (712 m^2^ g^−1^). In that cases the higher adsorption capacity can be related to the stronger interaction between DCF and TPEG. For the comparison with grape bagasse, higher surface area was observed for TPEG (47 vs 2 m^2^ g^−1^) and the higher adsorption capacity of TPEG could be also related to the higher surface of TPEG.

### Thermodynamic study

The best fit is represented by Eq. 20 with R^2^ = 0.9992.

20 $$ {\text{y}} = \, - {4665}.{\text{3x }} + { 17}.{173} $$

Therefore, the value of enthalpy and entropy obtained are 38.70 kJ mol^−1^ and 142.77 J mol^−1^ K^−1^, respectively. The driving force of the process is represented by the increase of entropy, associated to the increase in randomness at solid/solution interface during the adsorption process. The process is endothermic and a temperature above 271 K is required to ensure spontaneity of the process. Table 4 shows both the experimental and theoretical values of equilibrium constant ($\frac{{q}_{e}}{{C}_{e}}$) and free energy obtained from enthalpy and entropy values after the fitting.

**Table 4:** Thermodynamics evaluation of the adsorption process.

Temperature (K)	$\frac{{q}_{e}}{{C}_{e}}$ experimental (mg L)	$\frac{{q}_{e}}{{C}_{e}}$ theoretical (mg L)	ΔG^0^ experimental (kJ mol^−1^)	ΔG^0^ theoretical (kJ mol^−1^)
293	3.58	3.64	− 3.10	− 3.14
299	5.04	4.97	− 4.18	− 4.14
311	9.02	9.05	− 5.46	− 5.47

Equilibrium constant and free energy obtained from experimental and theoretical data. Experimental data obtained for DCF solution of 100 mg^−1^ L, 10 mg of adsorbent material, pH 1, stirring speed = 650 rpm and contact time = 10 min.

Experimental and theoretical data are comparable, confirming the fitting accuracy. Furthermore, the negative value of free energy confirms the spontaneity of the process.

The standard free energy is comparable with values reported in literature and indicates a physical adsorption of DCF on TPEG. The endothermicity of the process guarantees the possibility to improve the performance of the process by increasing the temperature of the batch. It indicates that the interaction between particles of DCF with itself is strongly than the interaction between DCF and TPEG surface and it can also explain the formation of multilayer of DCF on the surface of the TPEG. The major disorder caused by the breaking of bonding between of particles of DCF that were blocked casually on the surface of the TPEG is the driving force of the process. The comparison with other free energy of the adsorption of DCF on other material reported in literature demonstrated that the free energy of the adsorption on TPEG is one of the higher (considering the absolute value), therefore the process of adsorption is favored (higher free energy means higher equilibrium constant). The free energy calculated for the adsorption of DCF on TPEG was − 4.18 kJ mol^−1^, while values of − 4.30, 3.97 and − 0.99 kJ mol^−1^ are reported in literature for adsorption on CNT/HNO_3_^18^, grape bagasse^28^ and Zeolite modified with cetylpyridiumchloride^50^, respectively.

### Regeneration and reuse of TPEG

**Figure 10:**
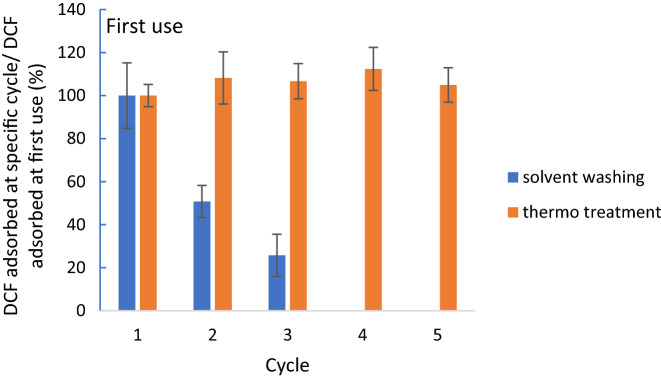
Performance of material after recovery treatment. Recovery performed by solvent washing with NaOH 0.2 M for 3 h at stirring speed of 800 rpm and thermo treatment at 105 °C for 2 h and 200 °C for 4 h. Adsorption step was done by using DCF 20 mg L^−1^, pH 1, stirring speed of 650 rpm, contact time of 10 min and 10 mg of adsorbent material. Standard deviation of each experimental data is also reported.

Figure 10 shows TPEG performance after the regeneration process.

The thermo recovery results to be the best way to recover TPEG because the amount of adsorbed DCF after each cycle is comparable to that of pure TPEG. After four cycles of thermo treatment the relative adsorption capacity results to be about 100%, proving that by heating the material all the DCF adsorbed on it is released and all the sites available for the adsorption interact with other molecules. The solvent washing is not a good way to recover the material because the high solubility of DCF in basic solutions is not enough to desorb DCF from TPEG. The relative q_e_ is about 50% and 25% after the first and second treatment, respectively. The reduction of relative q_e_ by using solvent washing is also justified by the loss of material during the separation water-TPEG. There is a technical limit due to the impossibility to do the filtration because the powder of the adsorbent material remains on the filter paper.

## Conclusions

In this study, TPEG was proposed as a good adsorbent material for DCF adsorption and removal from water. The morphological and structural analysis of TPEG demonstrates the presence of layers of sheet (n > 5) of graphene packed together, dimension of 24.02 nm of the crystallite and a surface area of about 47 m^2^ g^−1^. The point of strength of this material seems to be the possibility to reuse it without decreasing its adsorption performance by using a regeneration process not economically expensive (thermo-treatment). The adsorption results to be endothermic (38.70 kJ mol^−1^) and the driving force of the process is the increase of entropy (142.77 J mol^−1^ K^−1^) at the surface of the material caused by the increasing of disorder generated after the breaking of the interaction between the particles of DCF and their casual disposition on the surface of TPEG. The thermodynamics studies (free energy observed, ΔG^0^: − 4.18 kJ mol^−1^) suggest that physio-adsorption is involved. Pseudo-second order model regulates the kinetics of the process and Dubinin–Radushkevich model regulates the isotherm adsorption, therefore multilayer adsorption can be deducted as mechanism of adsorption. Values of adsorption capacity of about 400 mg g^−1^ was obtained by both the kinetics and isotherm experiments. The diffusion of DCF in the solution and into the pores of the material are faster than the “bonding” formation between DCF and active sites of TPEG. Even if the diffusion is faster than interaction on the active sites of TPEG, steric hindrance is involved caused by the formation of multilayer of DCF on the surface of TPEG. The pH influence on the adsorption process demonstrates that solubility of DCF deeply affects the adsorption process. Furthermore, the effect of initial concentration of DCF on adsorption capacity and the removal was evaluated and the ratio of 200 of [DCF]/[TPEG] was evidenced as the optimum ratio of maximum removal. By considering the experiments conducted in this work, it is possible to conclude that increase of surface area of TPEG and functionalization of TPEG surface can be useful to improve the rate of the process by increasing the rate of the interaction between DCF and active sites and to delay the pore blocking and increase the adsorption capacity. Further studies can be conducted on this material, in particular, its oxidation, magnetization or transformation of its morphology and then the evaluation of its adsorption capacity, the kinetics and the thermodynamics of the process of adsorption of same or new contaminants. Another useful study could be the use of TPEG as a filter to adsorb the same contaminant, so that the separation of treated water and TPEG is easier.

## Data Availability

The datasets used and/or analysed during the current study are available from the corresponding author on reasonable request.
